# Structural optimization method of rice precision direct seed-metering device based on multi-index orthogonal experimental

**DOI:** 10.3389/fpls.2023.1183624

**Published:** 2023-07-06

**Authors:** Hanqing Li, Lin Ling, Changkai Wen, Huaiyu Liu, Guangwei Wu, Xiaofei An, Zhijun Meng, Bingxin Yan

**Affiliations:** ^1^ Intelligent Equipment Research Center, Beijing Academy of Agriculture and Forestry Sciences, Beijing, China; ^2^ School of Electrical and Information, Northeast Agricultural University, Harbin, China; ^3^ State Key Laboratory of Intelligent Agricultural Power Equipment, Beijing, China

**Keywords:** rice, precision seed metering device, structural parameters, comprehensive optimization, matrix analysis

## Abstract

**Introduction:**

To improve the mechanization level of rice planting, a new type of direct seeding device for rice was designed. The device's structural properties will be crucial in determining its seeding performance. Structure optimization in the current seed metering device design process focuses on a single or few indexes, resulting in improved individual performance but imbalanced overall performance. Therefore, a structure optimization method of the direct seeding device based on a multi-index orthogonal experiment was proposed in this study.

**Methods:**

First, the DEM-MBD coupling method observed the factors and levels that affected the performance overall. Second, a test platform based on the electric drive control model was constructed, and a multi-index orthogonal test was devised. Finally, the structural parameters of the seed metering devices were optimized based on matrix analysis.

**Results:**

From the results, the primary and secondary levels of significance of factors were just as follows: hole diameter > hole number > adjustment angle. The following are the optimal parameters found by optimization analysis: the diameter of the hole was 12 mm, the number of holes was 10, and the adjustment angle was 80°. Validation tests were carried out and analyzed based on the optimal structural parameter combination. The qualification rate of seeds per hole, empty hole rate, average seed number, coefficient of variation of seed number, average hole spacing, and the variance coefficient of hole spacing are 93.07%, 0%, 9.39,14.04%, 22.84 cm, and 9.14%, respectively.

**Discussion:**

In comparison to traditional design and structural parameter optimization methods for rice precision seed metering device, this study not just to provides an optimization scheme for improving the overall performance of rice precision seed metering device, but also serves as a technical reference for the development and design of new rice precision seed metering device.

## 1 Introduction

Rice is the second largest food crop in the world, and about 50% of the world’s population lives on rice as a staple food (Jianbo et al., 2016). Increasing rice production is of far-reaching significance in solving the world food crisis (Xing et al., 2015a). Rice cultivation requires more labor and material resources than other crops (Farooq et al., 2011; Xing et al., 2015a). However, rice direct seeding technology can sow seeds directly into fields and cultivate them (Karayel, 2009; Gao et al., 2022; Kumar et al., 2022). This technology does not need traditional seedling raising and transplanting and is an important research direction of rice planting at present (Zhang et al., 2017; Pitoyo and Idkham, 2021; Xia et al., 2023).

The seed-metering device is an essential component for planting (Tang et al., 2022a; Tang et al., 2022b; Tang et al., 2023). The performance of the seed metering device has a direct impact on seeding quality (Gao et al., 2023). Singh et al. (2005) found that the shape and size of the pneumatic seed metering device can affect the qualified rate of seeding, which was verified by experiments. Karayel et al. (2006) discovered that the interaction of rotational speed and pressure might impact the qualification rate and miss-seeding index of sowing, as shown by an experiment and analysis of two factors and three indexes. Karayel et al. (2004) and Özmerzi et al. (2002)studied the vacuum degree of sowing different crops with the seed-metering device. They established the relationship equation between seed characteristics with four factors and a single index and determined the best structural parameters through experiments. Based on the response surface methodology, Yazgi et al. (2017) determined the influence of structural parameters such as suction hole diameter, suction hole number and rotational speed of the seed metering device on the working performance. At present, the seed metering device research technology seems reasonably developed, and it has been extensively employed in the real production of crops such as soybean, maize, wheat, and rape (Zhang et al., 2013).

Scholars have studied rice direct seed metering devices, but most optimized the structure based on a single or a few indexes. Xing et al. (2015b) designed a pneumatic seed metering device with an adjustable rice sowing rate. The number of holes, negative suction pressure, and rotating speed were shown to have a substantial influence on the pass rate and coefficient of variation of the seed-metering device. Zhang et al. (2018b) designed a combined hole seed metering device for the rice direct seeding machine. He found that the hole diameter, hole size, and rotating speed greatly affected the seed metering device’s qualified rate and coefficient of variation. Zhu et al. (2018) developed a slide hole-wheel precision seed metering device. He simulated and analyzed the seed metering device’s sowing performance at rotating speeds. The simulation method is unidirectional, and coupling simulation analysis is not used to study the device’s performance. At the same time, he assessed the performance using the qualification rate, missed sowing rate, and reseeding rate. Shun et al. (2020) designed a U-shaped cavity rice precision hole sowing seed metering device and found that the number and diameter of holes in the seed metering device can affect the qualified rate.

To conclude, the available study uses less than three indices to measure the performance of the seed metering device in the existing research (Guozhong et al., 2015; Liquan et al., 2017). Additionally, they all used single simulation approaches, resulting in a discrepancy between the experimental results and the empirical data. This situation should be attributed to the lack of multi-factor and multi-index research and the application of coupled simulation methods (Liquan et al., 2021; Wang et al., 2022). Because of the complexity of the working state of the seed metering device, the sowing performance is determined by multiple indexes and factors. Coupling simulation studies allow for the observation of device motion and provide a comprehensive understanding of mechanical changes within the device. As a result, device performance can be simulated and predicted with greater accuracy and thoroughness than with single-discipline simulation. Coupling simulation technology has already found wide application in the field of agricultural equipment (Zhu et al., 2018; Gao et al., 2021; LAI et al., 2022).

The structural parameters of the seed metering devices will influence their working quality, which in turn will influence the emergence and yield of rice seeds (Wei et al., 2017; Xing et al., 2021). Therefore, a structure optimization method of rice direct seed metering device based on a multi-index orthogonal test was proposed in this paper. Firstly, we determined the factors that affect the comprehensive performance of rice direct seed metering devices and their level through the coupling simulation analysis method. Secondly, we devised an orthogonal test based on several indices and constructed a test platform depending on the electric drive control model. Finally, we established a theoretical model of multi-index comprehensive optimization based on matrix analysis. The optimum structural parameters of the seed metering device were determined through the model, and the accuracy of the structural optimization method of the seed metering device based on a multi-index orthogonal test was verified qualitatively.

## 2 Materials and methods

### 2.1 Configuration and working principle of rice direct seed-metering device

**Figure 1:**
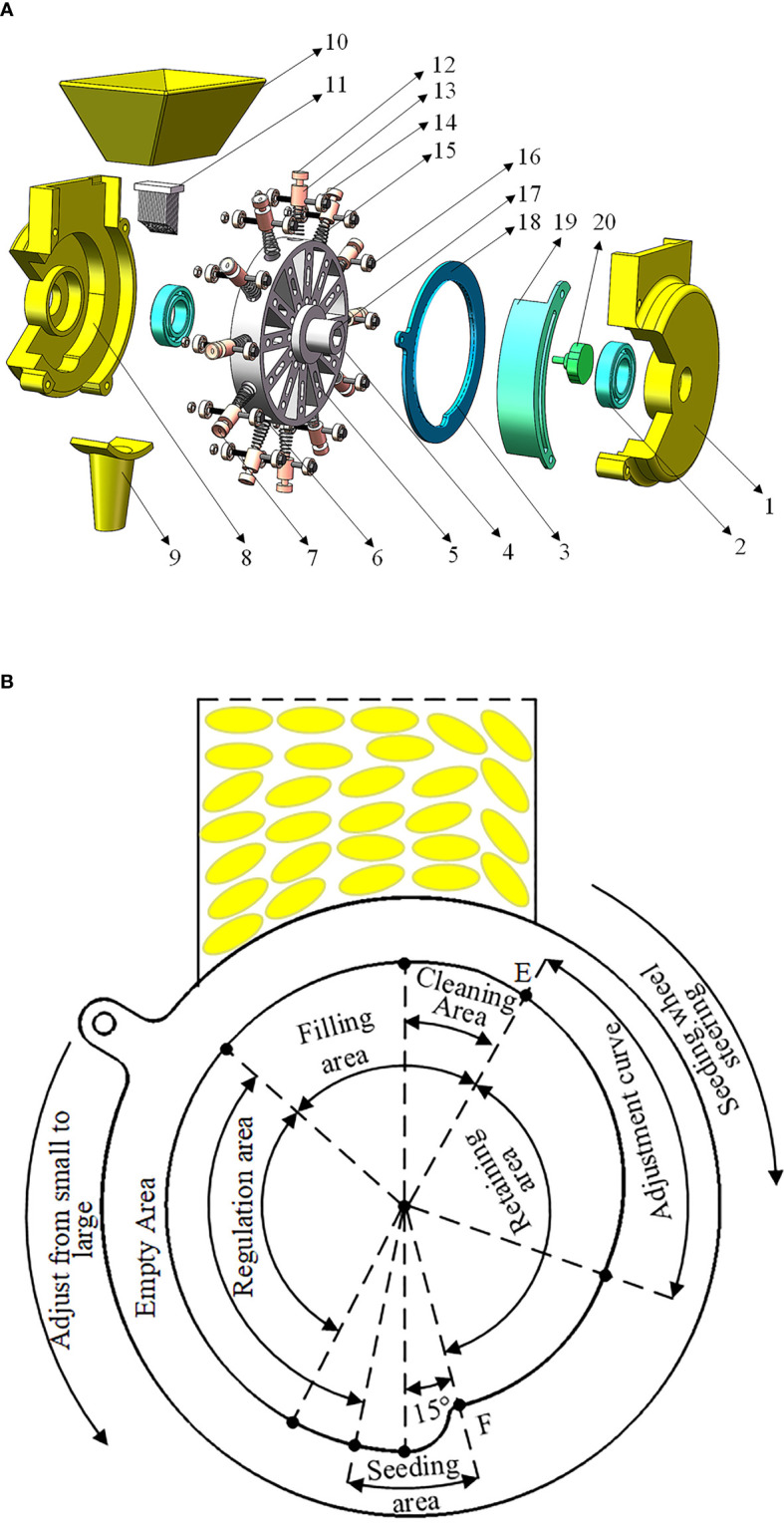
Diagram of structure and working principle of seed-metering device. **(A)** Axonometric drawing of seed metering device. **(B)** Adjusting mechanism and seeding process. 1. shell 2. supporting bearing; 3. inner track; 4. seeding wheel; 5. sliding groove; 6. hole; 7. guiding socket; 8. fixed track; 9. seed tube; 10. seedbox; 11. brush; 12. forced seeding mechanism; 13. push shaft; 14. rolling bearing; 15. Spring; 16. fixing bolt; 17. Seeding shaft; 18. seeding rate adjusting mechanism; 19. adjusting track; 20. adjusting handle.

The seed metering device designed in this study is shown in **Figure 1**, and it is primarily composed of a shell, seeding wheel, seeding shaft, brush, seed tube, forced seeding mechanism, seeding rate adjustment mechanism, and so on. The forced seeding mechanism is composed of a push shaft and a spring. The inner track of the sowing rate adjustment mechanism is composed of arc segments with different radii. The two tracks work together to drive the forced seeding mechanism up and down the sliding groove in the hole, changing the adjustable depth and completing the adjustment of varied seeding rates

The sowing rate adjustment mechanism should be adjusted to the theoretical sowing rate position before the seed metering device may function. Rice seeds complete the seed-filling process by virtue of gravity, seed pressure, and the rotation of the seed metering wheel. The seeds with an irregular posture that fall into the hole are cleaned by the brush and subsequently enter the retaining area, which is point E in **Figure 1B**. The adjusting depth of the hole is changed by the combined effect of the forced seeding mechanism and the seeding rate adjusting mechanism, allowing for varying amounts of seeds to be accommodated. When the seeding wheel passes through the retaining area and turns to a 15° angle directly below the seeding area, that is, at point F in **Figure 1B**, the forced seeding mechanism acts immediately under the action of double tracks, ejecting the seeds in the hole into the sowing ditch to complete sowing. The steering direction of the seeding wheel during the sowing process is opposed to the adjustment direction of the sowing rate from small to large, as shown in **Figure 1B**.

The seed metering device designed in this paper adopts the form of a gradual change in hole depth to realize sowing rate adjustment and adopts double tracks to realize the dynamic adjustment of the sowing rate. The adjustment method has the benefits of being easy to modify and having high accuracy. At the same time, the forced seeding mechanism effectively overcomes the issue of rice seed hole creation under card seed and multicast rates. Compared to existing rice direct seeding devices, this device has several advantages, including continuous adjustment of the sowing rate, a simple adjustment process, a more comprehensive adjustment range, and higher precision.

### 2.2 Optimization method of structural parameters of the seed-metering device based on multi-index orthogonal experiment

#### 2.2.1 Index selection and analysis of influencing factors of the seed-metering device performance

According to the results of related research (Yoo et al., 2005; Zhang et al., 2015; Wang et al., 2018), the qualification rate of seeds per hole y_1_, empty hole rate y_2_, average seeds per hole y_3_, coefficient of variation of seed number y_4_, average hole spacing y_5_, and the coefficient of variation of hole spacing y_6_ were selected as performance evaluation indexes in this study. Moreover, the empty hole rate is the ratio of the number of holes when the seed per hole is zero to the number of experimental groups. The seed metering device must meet the agronomic requirements, which include a qualification rate of seeds per hole of ≥ 85.0%, an empty hole rate not exceeding 5.0%, a coefficient of variation of seed number of ≤ 40.0%, a coefficient of variation of hole spacing of ≤ 30.0%, and an adjustable average hole spacing of 10~25 cm. The average seeds per hole is generally 3~8, but for cold areas in the north, the number of seeds in holes needs to be 8~10. The performance evaluation index is calculated as shown in Equation (1).


(1) $$\{\begin{array}{c} y_{1}=\frac{n_{1}}{N}\times 100\%\\ y_{2}=\frac{n_{2}}{N}\times 100\%\\ y_{3}=\frac{\sum_{i=1}^{N}N_{i}}{N}\times 100\%\\ y_{4}=\frac{S_{d}}{y_{3}}\times 100\%\\ y_{5}=\frac{\sum_{i=1}^{N}s_{i}}{N}\times 100\%\\ y_{6}=\frac{S_{d}^{'}}{y_{5}}\times 100\% \end{array}$$

Where *N* is the theoretical number of seed metering; *n*_1_ is the number of qualified holes for seed metering; *n*_2_ is the number of the empty hole; *N*_i_ is the seeds per hole in the i-th hole; s_d_ is the standard deviation of the seeds per hole; s_i_ is the hole spacing in the i-th hole; S_d_’ is the standard deviation of the hole spacing.

For the hole-type rice direct seed metering device, the probability of two seeds lying flat and one seed lying sideways entering the hole is the highest when the seeds are filled. However, the increasing hole diameter resulted in a difference in seed-filling number in every layer (Zhang et al., 2018a). So, the hole diameter can directly affect the seeds per hole, qualification rate, and coefficient of variation of the seed metering device. For different rice varieties and planting areas, the hole spacing must be 10 ~ 25 cm (adjustable) in direct seeding technology, and the optimum linear velocity of the seed metering plate is 0.2 ~ 0.35 m/s (Zhang et al., 2017; Xing et al., 2021). When the hole spacing is constant, increasing the number of holes reduces the rotating speed of the seed metering device. Therefore, the number of holes can directly affect the hole spacing and its coefficient of variation of the seed metering device. When the rotation angle of the seeding amount adjusting mechanism is different, the pre-filling seed area is different (Li et al., 2022). As the rotation angle of the seeding rate adjustment mechanism varies, so does the pre-filling seed area. If the pre-filling area is too small, it will lead to the inability to fill seeds, and then the empty hole rate of the seed metering device be increased.

This study conducted single-factor experiments based on the co-simulation approach to examine the impact of the structure parameters of the seed metering device on the performance index and identify the particular value range of the structure parameters. The effect of hole diameter, number, and adjustment angle on seed separator performance was investigated, and the range of variables was selected for orthogonal testing.

#### 2.2.2 Design of multi-index orthogonal experiment

The seed metering device’s single index test can only represent the main and secondary order of the structural characteristics, as well as the level’s importance (Xiaoling et al., 2010). The multi-index orthogonal test is based on the method of the orthogonal test. Based on single index optimization, the matrix analysis method is used to solve the comprehensive selection of the optimal configuration scheme of parameters (Li et al., 2021). Currently, the experimental indexes are less than three in most studies on the structural parameters of rice seed metering devices. The performance of the seed metering device can only be reflected from a broadening or local viewpoint, leading to a difference between the test results and the actual numbers. Therefore, the evaluation index in this paper can not only can meet the requirements of rice precision seeding from the qualified rate, but also demonstrate that the seed metering device has a broad seeding adjustment range and high accuracy based on the average number of holes and coefficient of variation, and that it meets the agronomic criteria for rice precision seeding based on the hole spacing and coefficient of variation. In this study, we plan to carry out the multi-index orthogonal test, which is shown in **Table 1**, and then obtain orthogonal test results through experiments.

**Table 1:** Schedule of multi-index orthogonal test.

Experimental number	Factors			
	*F*_a_	*F*_b_	*F*_c_	*F*_d_
1	1	1	1	1
2	1	2	2	2
3	1	3	3	3
4	2	1	2	3
5	2	2	3	1
6	2	3	1	2
7	3	1	3	2
8	3	2	1	3
9	3	3	2	1

F_a_, F_b_, F_c_, and F_d_ are different factors.

Optimization of structural parameters based on matrix analysis

The matrix analysis approach was utilized in this work to further evaluate and deal with the orthogonal test findings in order to thoroughly improve the structural parameters of the seed metering device under different indices. The method can solve the unreasonable configuration and selection of structural parameters of the seed metering devices under multiple indexes (Ruan et al., 2011). According to the data of the orthogonal test, a hierarchical data structure model composed of indexes, factors, and levels was constructed. The evaluation indexes, influencing factors, and factor levels of the seed metering device were regarded as the first, second, and third layers, respectively. In order to unify the evaluation indexes of the seed metering device, when ensuring the comparability of comprehensive optimization weights, we assume that there are m factors, each factor has n levels, and the average value of test indexes of factor *F*_i_ at the j level is k_ij_. When the evaluation index is large or small, K_ij_ equals k_ij_ or 1/k_ij_, respectively. The index evaluation matrix M is shown in Equation (2).


(2) $$M=\left[\begin{array}{ccc} K_{11} & \cdots & 0\\ \vdots & \ddots & \vdots \\ 0 & \cdots & K_{mn} \end{array}\right]$$

Secondly, we defined the factor layer matrix of the structural parameters of the seed metering device. When T_i_ equals $1/\sum_{i=1}^{n}K_{ij}$ , the factor layer matrix T is established, as shown in equation (3).


(3) $$T=\left[\begin{array}{ccc} T_{1} & \cdots & 0\\ \vdots & \ddots & \vdots \\ 0 & \cdots & T_{m} \end{array}\right]$$

Finally, we defined the horizontal layer matrix of structural parameters of the seed metering device. The range of factor *F*_i_ in the orthogonal test is S_i_. When S_i_ equals $s_{i}/\sum_{i=1}^{m}s_{i}$ , the horizontal layer matrix N is established, as shown in equation (4).


(4) $$N=\left[\begin{array}{c} S_{1}\\ S_{2}\\ \ldots \\ S_{m} \end{array}\right]$$

To sum up, the weight matrix γ of defining the structural parameters of the seed metering device is the product of the above three matrices, so the weight matrix γ is shown in equation (5).


(5) $$\gamma ={\left[\begin{array}{ccc} \gamma_{1} & \gamma_{2} & \begin{array}{cc} \cdots & \gamma_{n} \end{array} \end{array}\right]}^{T}$$

**Figure 2:**
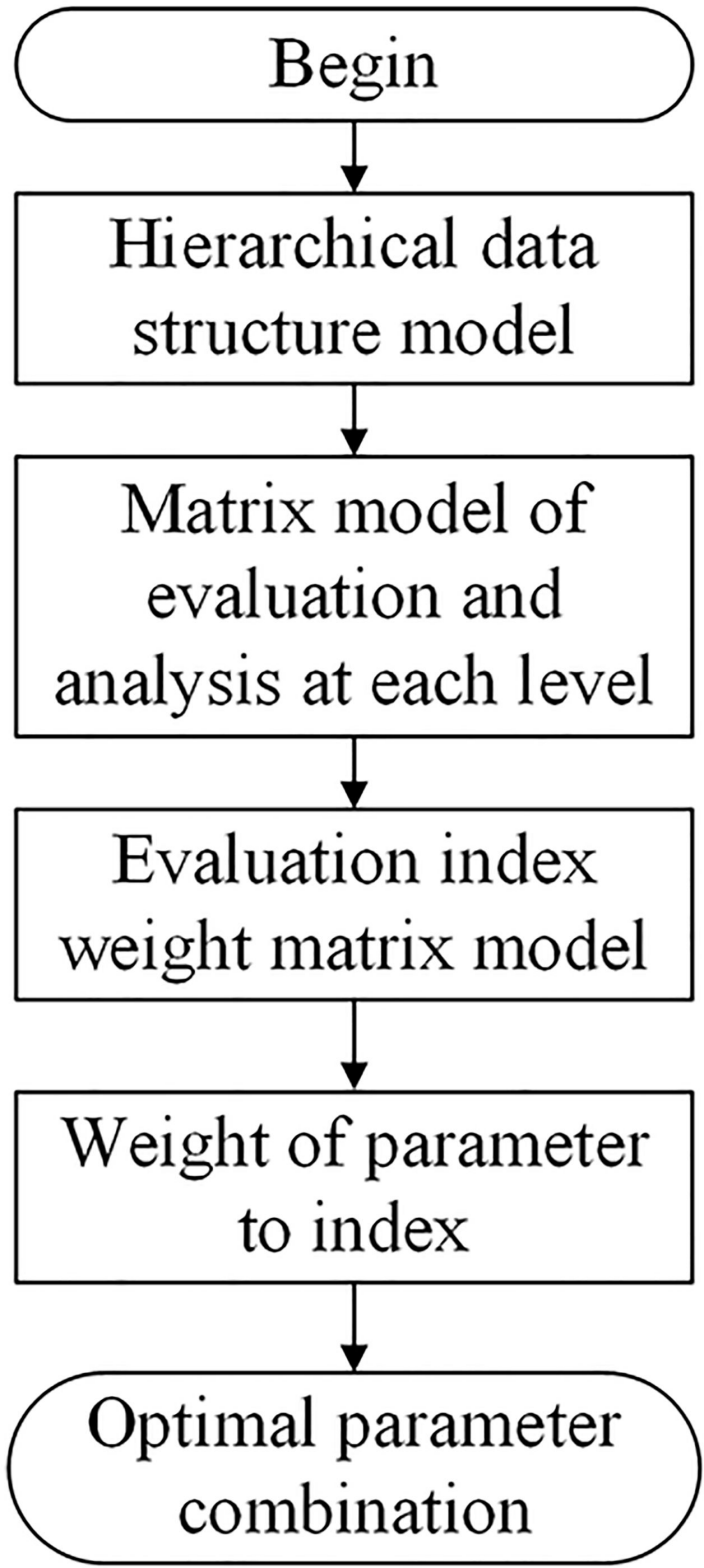
Parameter optimization flowchart.

The specific steps for multi-index structure parameter optimization based on matrix analysis is shown in **Figure 2**. Firstly, a hierarchical data structure model is constructed. Secondly, the matrix model of evaluation and analysis at each level is established. Finally, the weight matrix of the evaluation index is constructed. According to the theoretical analysis, we know the influence weight of the structural parameters of the seed metering device on each index. According to the weight, we can get the optimal structural parameters combination of the seed metering device and the primary and secondary order of the factors. Finally, after completing the multi-index parameter optimization, the effectiveness of the optimal parameter combination selected by the model was verified by experiments.

### 2.3 Experimental materials

Longken 58 rice seeds from Northeast China were selected as experimental materials. The mean values of the long axis, short axis, thickness, 1000-grain weight, moisture content, and sphericity of rice seeds were 6.98 mm, 3.29 mm, 2.35 mm, 24.84 g, 23.63%, and 54.14%, respectively. The rice seeds were washed and prepped before the experiment so that they could meet the direct planting criterion. According to the results of related research (Li et al., 2022), the comprehensive performance of the seed metering device decreased rapidly after the rotational speed exceeded 30r/min, and the field operating speed of the rice direct seeding machine was lower than 1.2 m/s. Therefore, the speed of the seed-metering wheel was set to 30 r/min, and the machine’s operating speed was set to 4 km/h.

## 3 Experiment

### 3.1 Simulation experiment

To ensure the accuracy of the test results and the simplicity of seeing the assessment index, the Discrete element method (DEM) and multi-body dynamics (MBD) software were used to build a simulation platform in this paper (Lai et al., 2022). The coupling simulation method was used to explore the influence of structural parameters on the performance index of the seed metering device. Finally, the factor level of critical structural parameters can be obtained by analyzing the simulation test results.

#### 3.1.1 Experimental design

The design processes are as follows, based on the DEM-MBD coupling simulation test: (1) the number of fixed holes is determined to be 12, the adjustment angle is determined to be 80, and the hole diameters are changed to 10, 12, 14 and 16 mm, respectively, and the simulation analysis were carried out; (2) Take the result of higher qualified rate in the first step as the typical value of analyzing the diameter of the hole. Based on typical values of fixed hole diameters and adjustment angles, the variables for the number of holes are set to 8, 10, 12, and 14, respectively. (3) The higher qualified rate and lower coefficient of variation in the first and second steps are taken as typical values for analyzing fixed adjustment angles. Based on the typical values of the fixed hole diameter and the number of holes, the experiments were carried out by changing the adjustment angles to 50, 60, 70, and 80°, respectively. According to the relevant research, results of the pre-test and rice varieties used in the test, when the diameter of the hole is 10, 12, 14, and 16, the number of seeds per hole is 5 ~ 7, 8 ~ 10, 15 ~ 17 and 22 ~ 24, respectively, which are regarded as qualified. The number of seeds per hole discharged by the seed metering device should be recorded, and every 250 holes should be divided into one group. Each group should be repeated three times, and the average value should be taken. The design of the test factor level is shown in **Table 2**.

**Table 2:** Levels of test factors.

Levels	Factors		
	Diameter of hole/mm	Number of holes	Adjust the angle/°
1	10	8	50
2	12	10	60
3	14	12	70
4	16	14	80

#### 3.1.2 Model construction based on DEM-MBD

**Figure 3:**
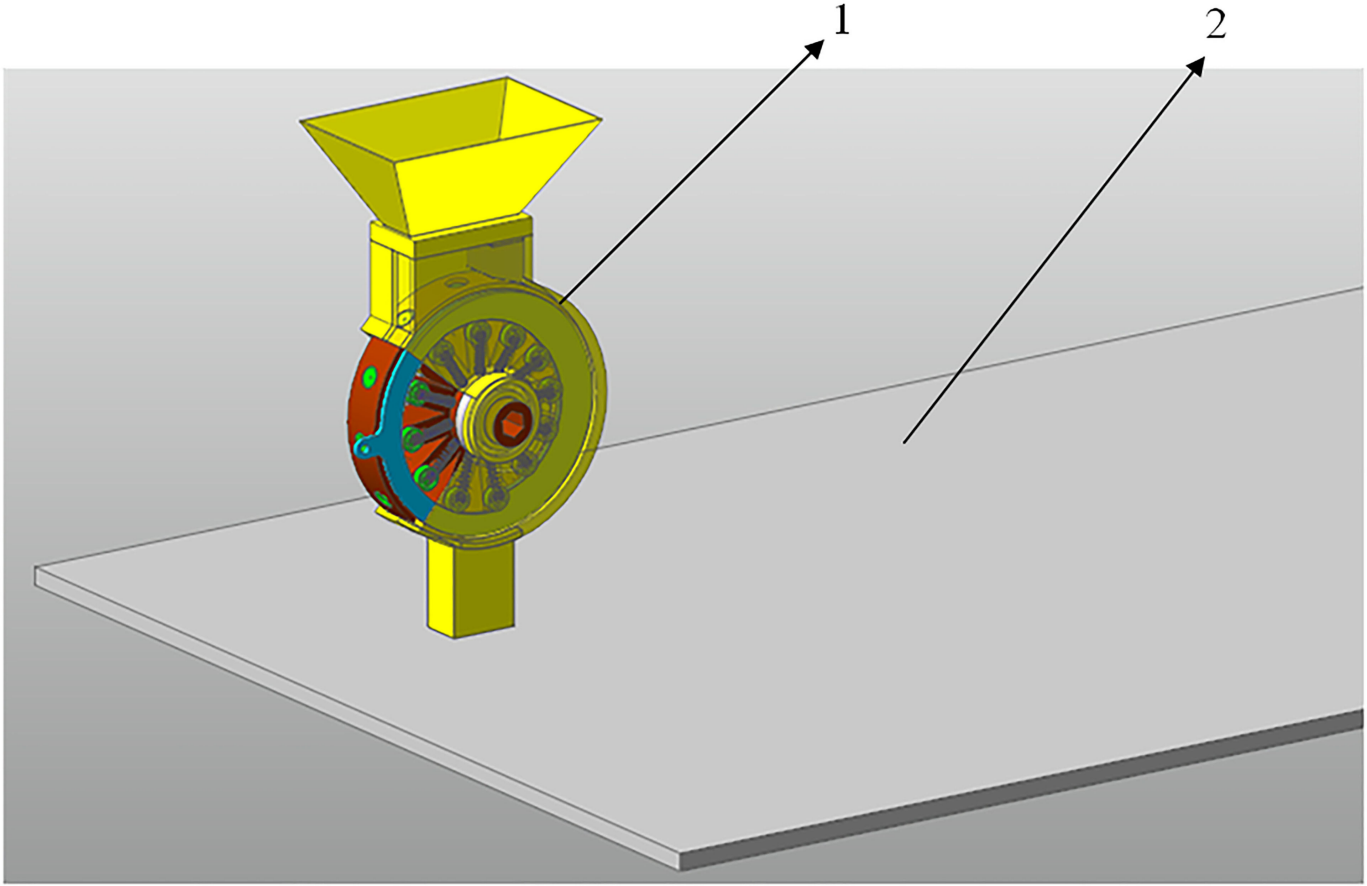
Model of the Seed-Metering Device in RecurDyn. 1. Seed-metering device; 2. Simulated ground.

The 3D model of the rice direct seed metering device was constructed by Solidworks 3D software, and the assembly model after the simplified structure was imported into RecurDyn, as shown in **Figure 3**. The simulation material property parameters are set as follows: The shell and seed metering wheel was made of ABS, and the Density of materials was set to 1.25 × 10^3^ kg/m^3^ by Density module (Ma et al., 2013; Jianbo et al., 2014). In order to improve the simulation efficiency and simplify the model, the ground running speed was set as the simulated running speed of agricultural machinery. Based on the working process of the seed metering device, IF (time-0.5: 0, 0, pi) was used as the driving function of the seed metering wheel (Lai et al., 2022). In other words, the rotational speed of the seed metering wheel is zero before 0.5 s, and the rotational speed of the seed metering wheel is 30r/min after 0.5 s. IF (time-0. 5: 0, 0, 1112) is used as the driving function of the simulated ground. In other words, the tool is stationary before 0.5 s and moves at 4 km/h after 0.5 s.

**Figure 4:**
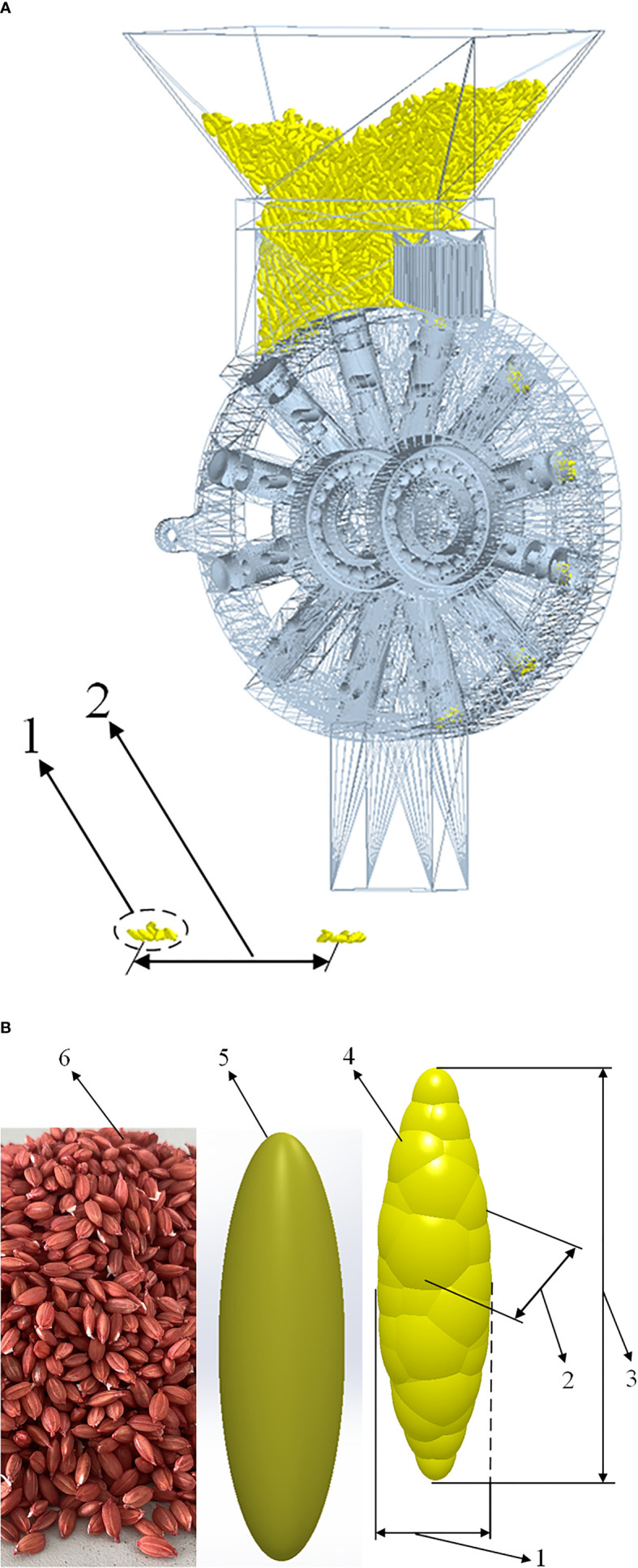
Discrete Element Model of Simulation. **(A)** Model of seed metering device in EDEM software. **(B)** Rice Seeds. 1. seeds per hole; 2. hole spacing; 3. short axis of rice seeds; 4. thickness of rice seeds; 5. The long axis of rice seed; 6. simulation model; 7. three-dimensional model; 8. real rice seed.

The discrete element model required for simulation is shown in **Figure 4**, and the EDEM model imported by the seed metering device is shown in **Figure 4A**. A three-dimensional model of rice seeds was established according to the shape of the rice seeds selected in the experiment. Then the model was imported into EDEM software, and the multi-spherical polymerized particle model of seeds was obtained through the fast-filling function of particles (Gao et al., 2021), as shown in **Figure 4B**.

Hertz-Mindlin’s non-slip model was used as the particle contact model in this research. The shell of the seed metering device and forced seeding mechanism were ABS injection molded parts, and the material of the brush was plastic. According to the relevant research results (Zhu et al., 2018), the contact parameters between particles and components are shown in **Table 3**.

**Table 3:** Parameters of discrete element simulation.

Parameters	Materials		
	Rice seeds	ABS	Brush
Poisson’s ratio	0.3	0.34	0.4
Shear modulus/Pa	1.82×10^8^	3×10^9^	1×10^8^
Density/(kg.cm^-3^)	1239	1250	1150
Recovery coefficient(With rice seed particles)	0.30	0.32	0.45
Static friction coefficient(With rice seed particles)	0.56	0.46	0.61
Coefficient of rolling friction(With rice seed particles)	0.01	0.01	0.02

In the simulation, the EDEM particle plant was set to generate 5000 seeds at a rate of 10000 seeds/s, and the total time to generate seed particles was set to 0.5 s. To maintain simulation continuity, a fixed time step of 1×10^-6^ s was established, which is about 25% of the Rayleith time step. To increase simulation efficiency, the entire simulation duration was set to 11s, with the rice seed generation time being less than 0.5 s.

### 3.2 Bench experiment

In the bench experiment, the motor drive was utilized to precisely adjust the working speed, simulating field sowing (Zhang et al., 2017). The rotating speed and working speed of the seed metering device are mathematically represented as shown in Equation (6).


(6) $$\frac{vt}{3.6}=\frac{R_{s}n_{s}dt}{60}=\frac{R_{m}n_{s}dt}{60i}$$

Where v is the running speed of the planter in km/h; T is the working time in s; R_s_ is the rotational speed of the seed metering device in r/min; R_m_ is the motor speed in r/min; n_s_ is the number of molded holes; d is the hole spacing in m; i is motor deceleration group.

Since the model is constructed at the same time, Equation (7) may be found by sorting the formula (6).


(7) $$d=\frac{50vi}{3R_{m}n_{s}}$$

According to equation (7), when the rotational and working speeds are constant, the hole spacing is only related to the number of holes. In other words, when the number of holes is 8, 10, 12 and 14, the theoretical hole spacing is 27.78, 22.22, 18.52, and 15.87 cm, respectively.

**Figure 5:**
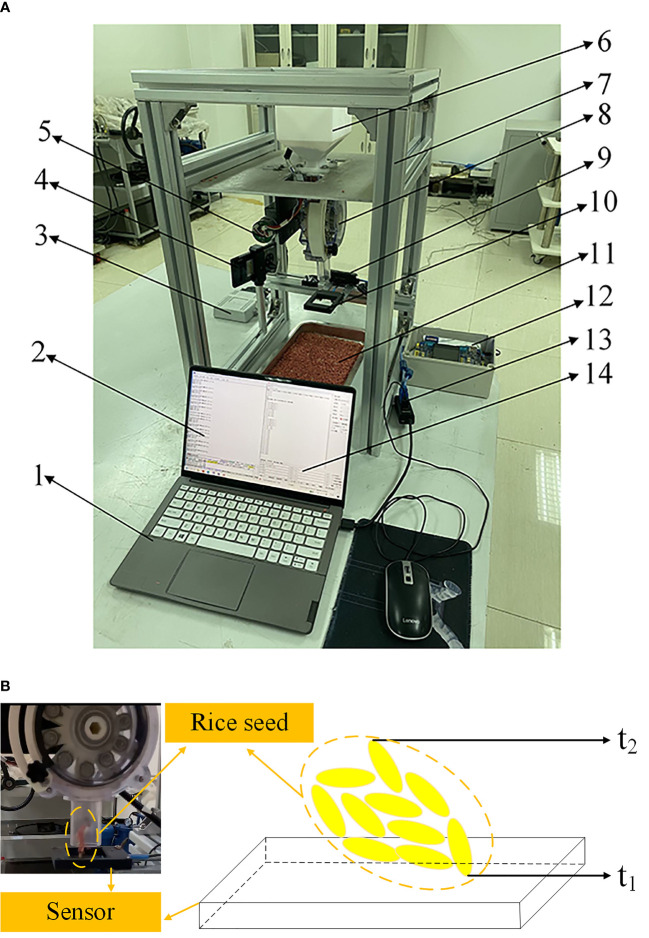
Test device and schematic diagram. **(A)** Experiment device. **(B)** Detection principle. 1. Computer terminal; 2. Sensor data acquisition interface; 3. Controller; 4. Image acquisition device; 5. Drive motor; 6. Seedbox; 7. Frame; 8. Seed metering device; 9. Optical fiber amplifier; 10. Optical fiber sensor; 11. Collecting device; 12. Data acquisition system; 13. Communication interface; 14. Speed control interface; The first seed in the first hole enters the sensor at t_1_, the last seed at t_2_; The first seed in the second hole at t_3_, and the last seed at t_4_.

In this paper, the sowing test platform was set up as shown in **Figure 5**. The experimental site is the Agricultural Machinery Equipment Laboratory of the National Research Center for Intelligent Agricultural Equipment (Beijing, China). The test platform mainly comprises a rack, seed metering device, computer terminal, data acquisition device, camera acquisition device, and other components. The seed metering device is fixed on the frame, and the output shaft of the driving motor is connected to the seeding shaft. The data is transferred to the controller through the computer terminal’s rotational speed control interface, and the rotational speed of the seed metering device is precisely regulated using the control model specified by equation (7). The detailed parameters of the experimental platform are in Section 2.3 of this paper. The image acquisition device can record the seed-dropping process of the seed tube, and the number of rice seeds per hole can be recorded through data processing after the experiment. For the convenience of later data processing and the authenticity of data, the performance parameters of the image acquisition device are adjusted to 7680 f/s for data acquisition.

The optical fiber sensor was installed under the seed tube to detect the time between seeds in each hole. A fiber amplifier was used to amplify the sensor’s signal and convert the optical signal into the electrical signal. The data acquisition system was used to collect the sensor’s output data, transmit the data to the computer terminal through the communication interface, and then obtain the corresponding evaluation index after post-processing. The sensor detection principle is shown in **Figure 5B**. The time when the first seed in the first hole enters the sensor is recorded as t_1_, and when the last seed leaves, the sensor is recorded as t_2_. Therefore, we can make the following definition: the time T_1_ of the first hole is (t_2_+t_1_)/2, the time T_2_ of the second hole is (t_4_+t_3_)/2, and the interval time between the first point and the second hole is the difference between T_2_ and T_1_. The response speed of the sensor selected in this test is 2 μs, and the detection accuracy of the sensor meets the requirements through the pre-test. Combined with the derivation of relevant theoretical models and the pre-test results (Zhang et al., 2016; Li et al., 2022), the interval between rice seeds in each hole is far less than between holes. Therefore, the influence of the interval time between rice seeds in each hole on the results of this study can be ignored.

As the sensor collects excessive data in each group of tests, the data processing procedure will be briefly described by considering the original data of one hole distance collected by the sensor, as shown in **Table 4**. When a seed enters the sensor, it triggers the sensor to a low level. Conversely, when no seed enters the sensor, it triggers the sensor to a high level. Based on the sensor’s working principle, the data acquisition system records the time when the rice seed triggers the high and low levels. The intermediate time T_1_ of the first hole and T_2_ of the second hole can be calculated, and the time interval between the two holes can be further determined. Utilizing the time interval and simulated speed in the test, the value of the first hole distance can be calculated. Similarly, all the hole distance values in the experiment can be obtained using this method.

**Table 4:** Original sensor data (One hole spacing).

Number	Record time	Level state	Intermediate time	Time between holes	Hole spacing/m
1	18:32:33.911 (t_1_)	Low	18:32:33.928 (T_1_)	0.204	0.227
2	18:32:33.944 (t_2_)	High			
3	18:32:34.121 (t_3_)	Low	18:32:34.132 (T_2_)		
4	18:32:34.143 (t_4_)	High			

## 4 Results and analysis

### 4.1 Results of single factor simulation analysis

The influence of hole diameter, number, and adjusting angle on the performance of the seed metering device was evaluated by a single factor in section 2.2.1, and the analysis results may determine the value range of factors for the orthogonal test. The following are the particular research and analysis.

#### 4.1.1 Influence of hole diameter on seed metering device performance

**Figure 6:**
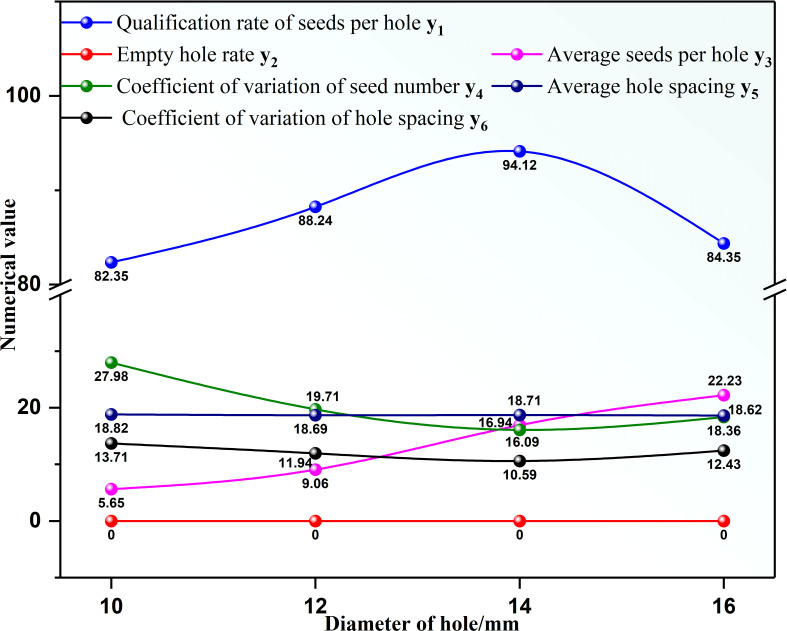
Effect of different hole diameters on the seed-metering device performance.

Different hole diameters lead to a different number of seeds filled in each layer, affecting the seed metering device’s performance index. The analysis of specific influence degrees is as follows. According to the results of single factor analysis, the hole diameter range can be accurately determined, and the factor level can be determined for the orthogonal test. The influence of different hole diameters on the performance of the seed metering device is shown in **Figure 6**.

As shown in **Figure 6**, with the increase of hole diameter, the qualification rate of seeds per hole y_1_ first increases and then decreases, the coefficient of variation of seed number y_4_ first decreases and then increases, the coefficient of variation of hole spacing y_6_ first decreases and then increases, the average seeds per hole y_3_ shows an increasing trend, the empty hole rate y_2_ is zero, and the average hole spacing y_5_ is almost unchanged. When the hole diameter was 14 mm, the qualification rate of seeds per hole was 94.12%, the coefficient of variation of seed number was 16.09%, and the coefficient of variation of hole spacing was 10.59%. When the hole diameter was 10 mm, the qualification rate of seeds per hole was 82.35 (the lowest), the coefficient of variation of seed number was 27.98% (the highest), and the coefficient of variation of hole spacing was 13.71% (the highest). Therefore, the hole diameters of 12, 14, and 16 mm were selected as the orthogonal test factor levels in the following experiments. According to section 3.1.1, the hole diameter with the higher qualification rate is the typical value for subsequent single-factor analysis. The diameter of the hole was fixed at 14 mm in the subsequent single-factor test.

#### 4.1.2 Influence of the number of the hole on the seed metering device performance

**Figure 7:**
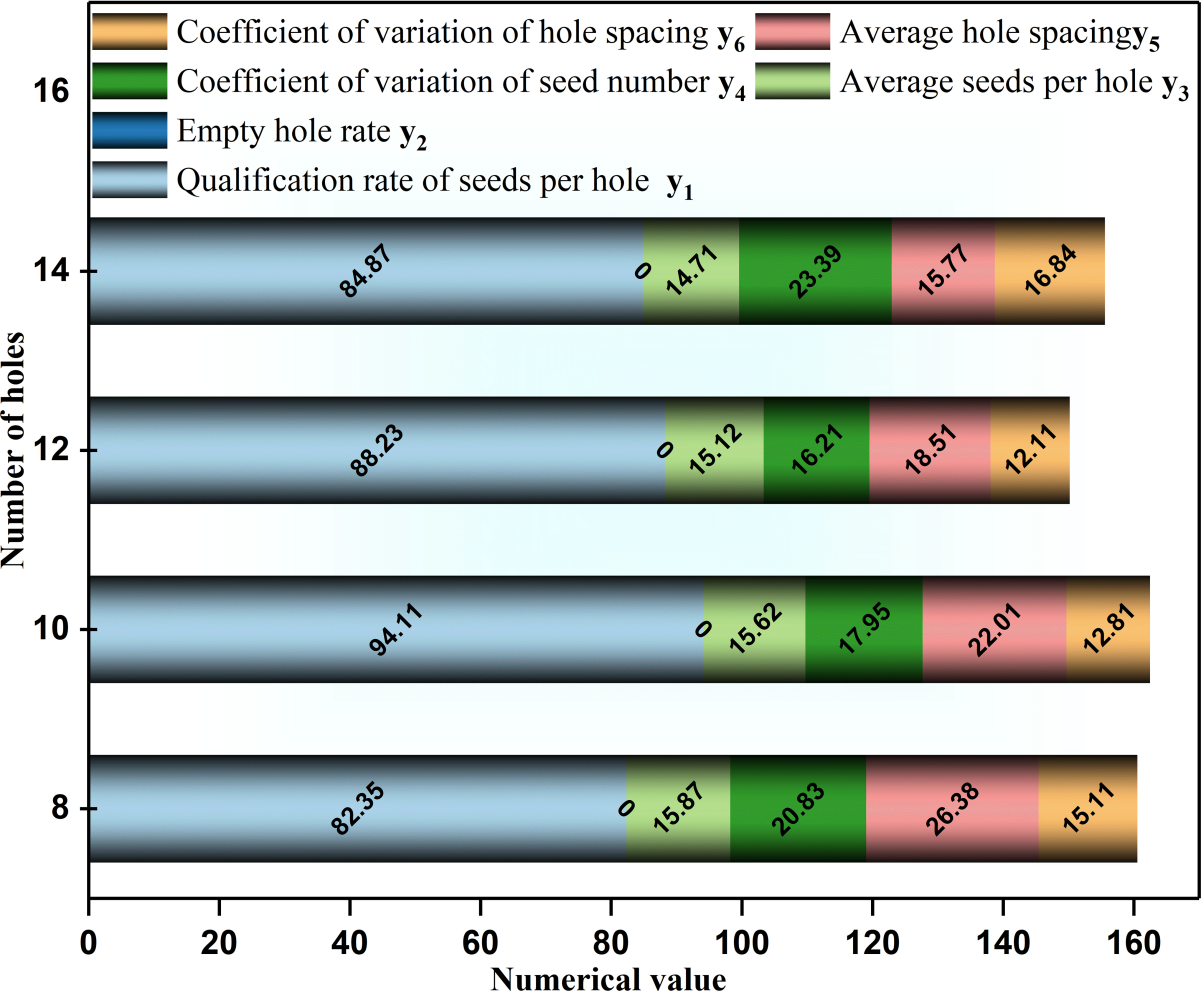
Influence of different holes number on the seed-metering device performance.

When the seed metering device’s rotational speed is constant, the performance of the seed metering device can be improved by changing the number of holes. The specific influence of the number of holes on the performance of the seed metering device is as follows. From the analysis results, we can determine the factor level value of the orthogonal test, and the influence of different hole numbers on the performance of the seed metering device is shown in **Figure 7**.

The results show that with the increase of the number of holes, the qualification rate of seeds per hole y_1_ first increases and then decreases, the coefficient of variation of seed number y_4_ first decreases and then increases, the coefficient of variation of hole spacing y_6_ first decreases and then increases, the average seeds per hole y_3_ and the average hole spacing y_5_ gradually decrease, and the empty hole rate y_2_ is zero. When the number of holes was 10, the qualification rate of seeds per hole was 94.11%, and the coefficient of variation of seed number was 17.95%. When the number of holes was 12, the qualification rate of seeds per hole was 88.23%, slightly lower than that when the number of holes was 10 (94.11%). However, the coefficient of variation of seed number (16.21%) is better than that when the number of holes was 10. According to section 3.1.1, the number of holes with the higher qualification rate and lower coefficient of variation of seed number was regarded as the typical value of subsequent single-factor analysis. Therefore, in the subsequent single-factor test, the number of holes was determined to be 12. When the number of holes was 8, the qualification rate of seeds per hole was 82.35% (the lowest). For precision direct seeding technology of rice, the qualification rate of seeds per hole can directly reflect whether the seed metering device meets the requirements of precision direct seeding. Therefore, the number of holes 10, 12, and 14 was selected as the orthogonal test factor level in the subsequent test.

#### 4.1.3 Influence of adjusting angle on seed metering device performance

**Figure 8:**
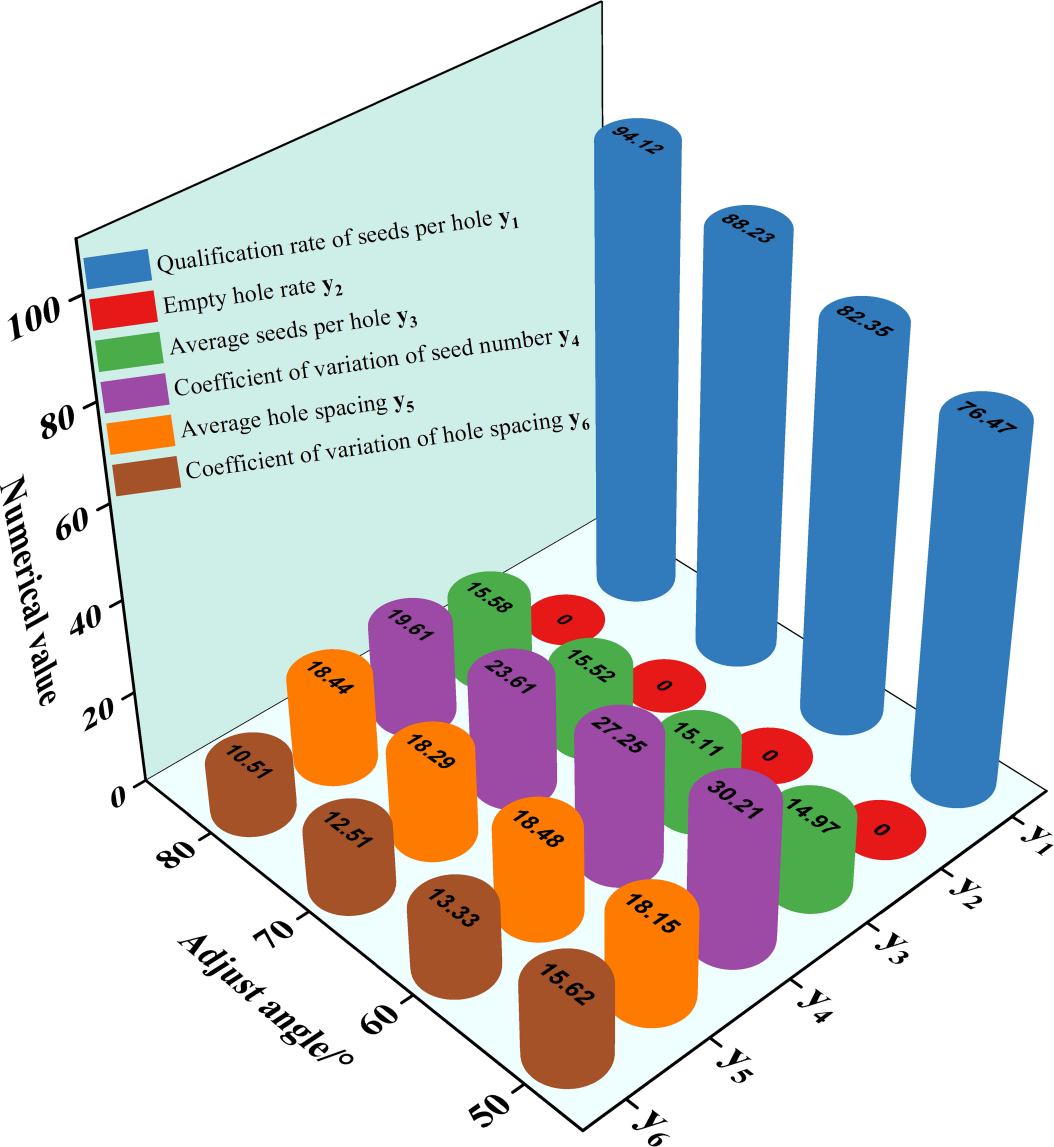
Influence of different adjustment angles on the seed-metering device performance.

Different adjustment angles indicate different pre-filling seed areas. Too small pre-filling seed areas can affect the filling seeds of the seed metering device, and then affect the sowing performance of the seed metering device. The specific effects are explained as follows. The influence of different adjustment angles on the performance of the seed metering device is shown in **Figure 8**.

Through the analysis of **Figure 8**, it can be seen that with the increase of adjustment angle, the qualification rate of seeds per hole y_1_ shows an upward trend, the coefficient of variation of seed number y_4_ and the hole spacing y_6_ shows a downward trend, the average seeds per hole y_3_ and the average hole spacing y_5_ show an increasing trend, and the empty hole rate y_2_ is zero. When the adjustment angle was 50, the qualification rate of seeds per hole (76.47%) was lower than 80%. According to the results of related research, if the qualification rate of seeds per hole is less than 80%, it will not meet the requirements of precision sowing. Therefore, the adjustment angles of 60°, 70°, and 80° were selected as the factor levels of the orthogonal test in the subsequent test.

### 4.2 Analysis results of orthogonal experiment

According to Section 2.2, it is necessary to carry out the orthogonal test based on the single-factor test results after completing it. The orthogonal test results of structural parameter optimization of the seed metering device are shown in **Table 5**.

**Table 5:** Results of orthogonal test.

Number	Structural parameters				Evaluation index					
	A	B	C	D	y_1_/(%)	y_2_/(%)	y_3_/(grain)	y_4_/(%)	y_5_/(cm)	y_6_/(%)
1	1 (12)	1 (10)	1 (60°)	1	80.73±1.24	0.00±0.00	8.95±0.56	18.88±2.32	21.63±0.13	15.79±2.37
2	1 (12)	2 (12)	2 (70°)	2	81.41±1.12	0.13±0.00	8.97±0.71	17.47±2.01	18.23±0.17	12.37±1.97
3	1 (12)	3 (14)	3 (80°)	3	85.61±1.36	0.00±0.00	9.18±0.62	14.84±2.67	15.87±0.15	12.98±2.08
4	2 (14)	2 (12)	3 (80°)	1	94.47±1.78	0.00±0.00	16.09±0.87	14.93±2.07	18.67±0.23	10.62±1.86
5	2 (14)	3 (14)	1 (60°)	2	85.07±1.23	0.37±0.00	15.82±1.04	23.12±1.98	16.21±0.14	17.23±2.46
6	2 (14)	1 (10)	2 (70°)	3	91.27±1.38	0.00±0.00	15.91±0.82	17.91±1.56	21.98±0.47	8.28±2.12
7	3 (16)	3 (14)	2 (70°)	1	82.73±1.35	0.00±0.00	22.16±1.33	23.81±1.98	15.99±0.36	17.01±2.52
8	3 (16)	1 (10)	3 (80°)	2	84.73±1.42	0.00±0.00	22.23±1.51	19.35±1.56	21.55±0.24	10.04±2.22
9	3 (16)	2 (12)	1 (60°)	3	80.07±1.47	0.00±0.00	22.12±1.63	28.14±2.78	18.44±0.36	22.74±2.55

#### 4.2.1 Range analysis

**Figure 9:**
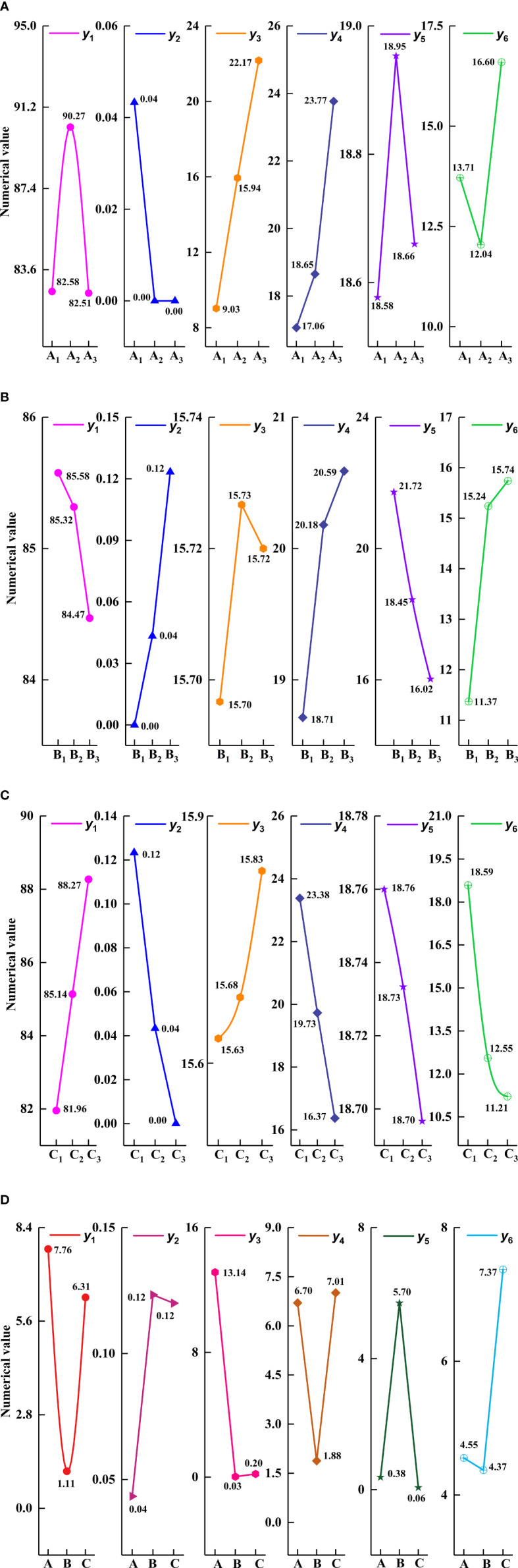
Range analysis of single index. **(A)** The influence of hole diameter on the performance index. **(B)** The influence of the hole number on the performance index. **(C)** The influence of adjustment angle on a performance index. **(D)** Range analysis of factors on a performance index. y_1_ is the qualification rate of seeds per hole; y_2_ is the empty hole rate; y_3_ is the Average seeds per hole; y_4_ is the coefficient of variation of seed number; y_5_ is the average hole spacing; y_6_ is the coefficient of variation of the hole spacing.

The range analysis of the orthogonal test results of the seed-metering device is shown in **Figure 9**. From the order of primary and secondary factors, the main parameters affecting the qualification rate of seeds per hole were hole diameter and adjustment angle. The main parameters affecting the empty hole rate are the number of holes and the adjustment angle. The hole diameter was the main parameter affecting the average number of seeds per hole. The main parameters affecting the coefficient of variation of seed number were hole diameter and adjustment angle. The main parameter affecting the average hole spacing was the number of holes. The adjustment angle was the main factor affecting the coefficient of variation of the hole spacing. Parameters were selected based on the higher qualified rate of hole number. The optimized horizontal combination of structural parameters of the seed metering device is A_2_B_1_C_3_. That is, the diameter of the hole was 14 mm, the number of the hole was 10, and the adjustment angle was 80°.

Similarly, the optimum level combinations of the empty hole rate, coefficient of variation of seed number, and hole spacing were A_2_B_1_C_3_, A_1_B_1_C_3,_ and A_2_B_1_C_3_, respectively. The average seeds per hole and hole spacing should be selected under the premise of a small coefficient of variation. The optimal level combination of average seeds per hole is A_1_B_1_C_3_, and the optimal level combination of average hole spacing is A_2_B_1_C_3_.

#### 4.2.2 Variance analysis

**Figure 10:**
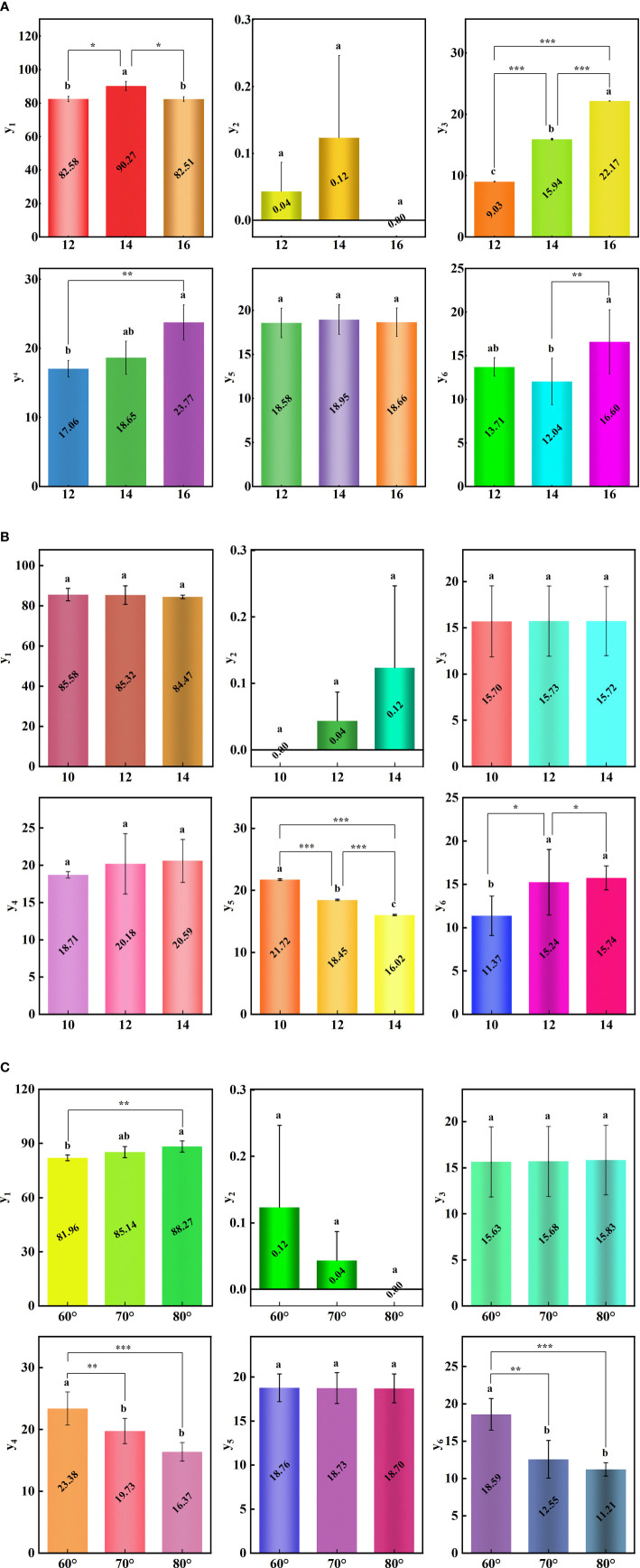
Variance analysis of single index. **(A)** Variance analysis of hole diameter. **(B)** Variance analysis of the hole number. **(C)** Variance analysis of adjustment angle. y_1_ is the qualification rate of seeds per hole; y_2_ is the empty hole rate; y_3_ is the average seeds per hole; y_4_ is the coefficient of variation of seed number; y_5_ is the average hole spacing; y_6_ is the coefficient of variation of hole spacing; * indicates that the difference is significant at 0.05 level; ** indicates that the difference is significant at 0.01 level; *** indicates that the difference is significant at 0.001 level.

The variance analysis of the orthogonal test results of the seed metering device is shown in **Figure 10**. The analysis shows that the hole diameter and adjustment angle can significantly influence the qualification rate of seeds per hole. The hole diameter can highly significantly affect the average number of seeds per hole. The hole diameter and adjustment angle significantly influence the coefficient of variation of a seed number. The number of holes can highly significantly influence the average hole spacing. The diameter and number of holes and the adjustment angle significantly influence the coefficient of variation of the hole spacing. However, the diameter, number of holes, and adjustment angle have no significant influence on the empty hole rate. The diameter of the hole has no significant influence on the average hole spacing. The number of holes has no significant influence on the qualification rate of seeds per hole, the average seeds per hole, and the coefficient of variation of a seed number.

### 4.3 Results of parameter optimization

The matrix analysis method was used to optimize the structure parameters of the seed metering device, which solves the unreasonable problem of multi-factor optimization under multi-index. According to the theoretical analysis in section 2.3.3, the weight matrix of each evaluation index of the seed metering device can be calculated as shown in equations (8)–(13).


(8) $$\gamma_{y_{1}}={\left[\begin{array}{ccc} 0.1653 & 0.1807 & \begin{array}{ccc} 0.1652 & 0.0224 & \begin{array}{ccc} 0.0224 & 0.0241 & \begin{array}{cc} 0.1335 & \begin{array}{cc} 0.1387 & 0.1438 \end{array} \end{array} \end{array} \end{array} \end{array}\right]}^{T}$$

(9) $$\gamma_{y_{2}}={\left[\begin{array}{ccc} 0.0000 & 0.0000 & \begin{array}{ccc} 0.1494 & 0.4252 & \begin{array}{ccc} 0.0001 & 0.0000 & \begin{array}{ccc} 0.0000 & 0.0001 & 0.4252 \end{array} \end{array} \end{array} \end{array}\right]}^{T}$$

(10) $$\gamma_{y_{3}}={\left[\begin{array}{ccc} 0.4977 & 0.2821 & \begin{array}{ccc} 0.2028 & 0.0007 & \begin{array}{ccc} 0.0007 & 0.0007 & \begin{array}{ccc} 0.0051 & 0.0051 & 0.0050 \end{array} \end{array} \end{array} \end{array}\right]}^{T}$$

(11) $$\gamma_{y_{4}}={\left[\begin{array}{ccc} 0.1634 & 0.1491 & \begin{array}{ccc} 0.1173 & 0.0452 & \begin{array}{ccc} 0.0394 & 0.0386 & \begin{array}{ccc} 0.1244 & 0.1474 & 0.1777 \end{array} \end{array} \end{array} \end{array}\right]}^{T}$$

(12) $$\gamma_{y_{5}}={\left[\begin{array}{ccc} 0.0206 & 0.0202 & \begin{array}{ccc} 0.0205 & 0.2628 & \begin{array}{ccc} 0.3094 & 0.3562 & \begin{array}{ccc} 0.0034 & 0.0034 & 0.0034 \end{array} \end{array} \end{array} \end{array}\right]}^{T}$$

(13) $$\gamma_{y_{6}}={\left[\begin{array}{ccc} 0.0942 & 0.1073 & \begin{array}{ccc} 0.0779 & 0.1086 & \begin{array}{ccc} 0.0810 & 0.0785 & \begin{array}{ccc} 0.1093 & 0.1619 & 0.1812 \end{array} \end{array} \end{array} \end{array}\right]}^{T}$$

The index of the qualification rate of seeds per hole should be maximized to ensure the best overall performance of the seed metering device. In contrast, the empty hole rate, the coefficients of variation of the seed number, and the hole spacing should be as low as possible. Combined with the structural parameters and experimental conditions, the calculated theoretical seeds per hole and hole spacing all meet the agronomic requirements. Therefore, the average seeds per hole and the hole spacing should be the parameters when the corresponding coefficient of variation is the smallest. That is, the average seeds per hole and the hole spacing should be the smallest. In summary, if the total weight matrix of multi-index evaluation is the average of a single index matrix, then there is a total weight matrix, as shown in Equation (14).


(14) $$\gamma_{T}={\left[\begin{array}{ccc} 0.1569 & 0.1233 & \begin{array}{ccc} 0.1222 & 0.1440 & \begin{array}{ccc} 0.0758 & 0.0830 & \begin{array}{ccc} 0.0626 & 0.0761 & 0.1560 \end{array} \end{array} \end{array} \end{array}\right]}^{T}$$

We can observe from the total weight matrix that A_1_ is the highest weight of factor A, indicating that the first level of hole diameter in the structural parameter of the seed metering device has the most effect on the multi-index test results. Similarly, the first level in factor B has the most significant influence on the results of the multi-index test, and the third level in factor C has the most significant influence on the results of the multi-index test. Therefore, the optimal factor level combination of structural parameters of the seed-metering device is A_1_B_1_C_3_. The hole diameter, number of holes, and adjustment angle was 12 mm, 10, and 80°, respectively. The primary and secondary order of structural parameter effect on the complete assessment index is A > B > C.

### 4.4 Verification experiment

**Figure 11:**
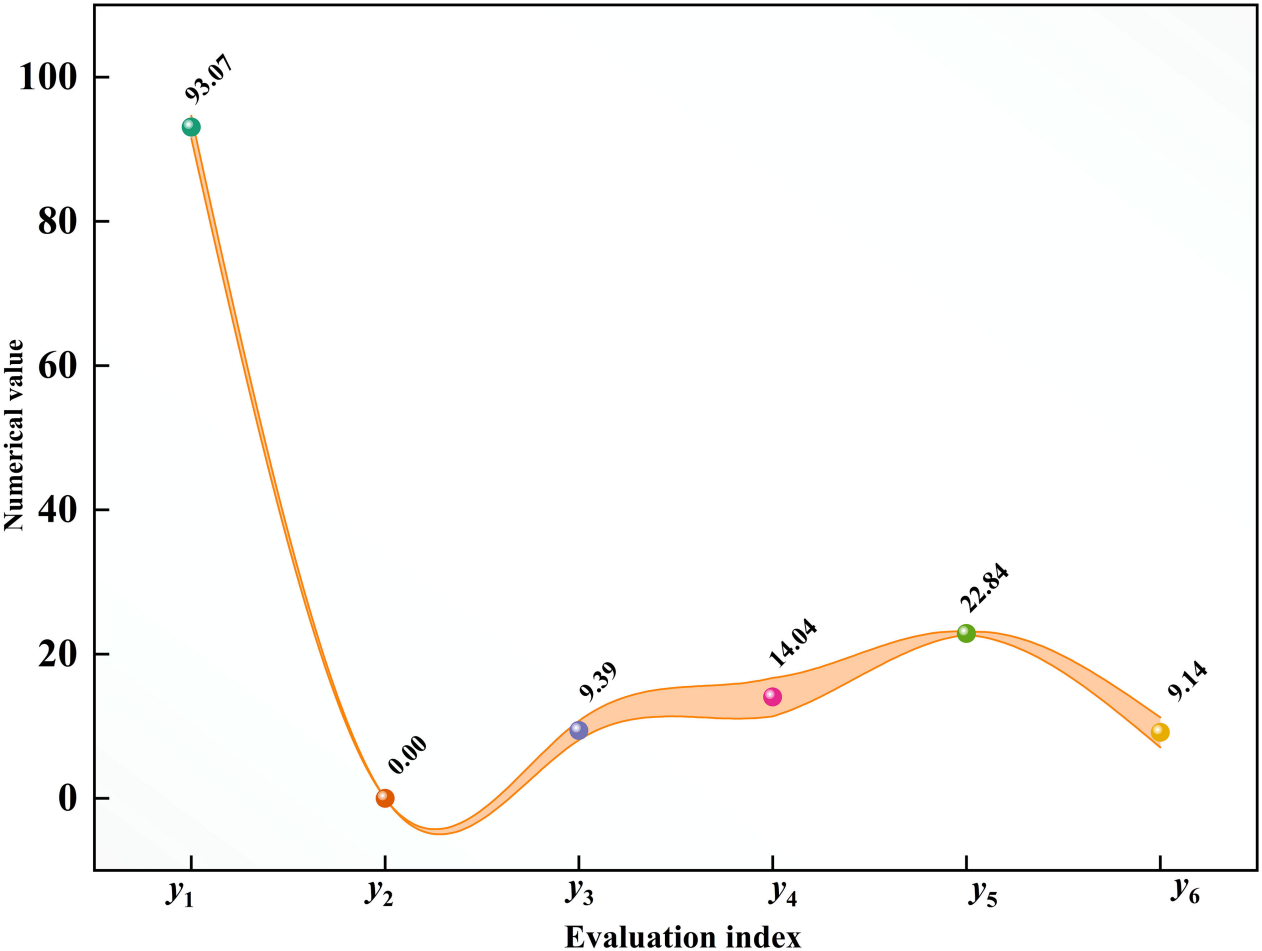
Results of optimal combination test. y_1_ is the qualification rate of seeds per hole; y_2_ is the empty hole rate; y_3_ is the average seeds per hole; y_4_ is the coefficient of variation of seed number; y_5_ is the average hole spacing; y_6_ is the coefficient of variation of the hole spacing.

The verification experiment occurred under identical test conditions using the ideal combination of structural characteristics, the experiment was repeated three times, as well as the average value was taken. The experimental results are shown in **Figure 11**. Based on the comprehensive optimization method proposed in this paper, the specific research methods are described in Section 2.2.2. The optimization objective is to improve the overall performance of the seed metering device based on the premise of a higher percentage of the qualification rate of seeds per hole and smaller values of other evaluation indexes. The weight matrix of each factor can be obtained through theoretical analysis, and the total weight matrix can be calculated by using the weight matrix of each factor. The greater the weight, the greater the influence of the factor level on the index, that is, the better the performance of the seed metering device. Based on the evaluation standard of the weight ratio, the optimal combination of structural parameters of the seed metering device can be obtained. The seed metering device under the optimal combination of structural parameters, the qualification rate of seeds per hole, empty hole rate, average seeds per hole, coefficient of variation of seed number, average hole spacing, and the coefficient of variation of hole spacing was 93.07%, 0%, 9.39, 14.04%, and 22.84 cm, 9.14%, respectively. According to the condition of the verification test and the mathematical control model designed in this paper, the theoretical hole spacing should be 22.22 cm. Therefore, the error between the verification test results and the theoretical values is 2.79%.

## 5 Discussion

The connection between the evaluation index and each component was analyzed to determine the variables that impact the performance index of the seed metering device. To begin, the single-factor test serves to establish the value range of each structural parameter. The orthogonal test would then be run to determine the best combination of structural parameters. Finally, the validity of the modified structural parameters was tested, and the seed metering mechanism was fully evaluated. Following is a discussion of the main test results of a comprehensive analysis:

1) The diameter of the hole affects the performance of the seed metering device (**Figure 6**). The number of seeds entering the hole rises as the diameter of the hole increases, resulting in the probability of irregular movement of seeds in the hole, impacting the qualification rate of seeds per hole and the coefficient of variation of a seed number. The number of holes has an effect on the performance of the seed metering device (**Figure 7**). If the number of holes is too small, the seed filling time will be too excessive, affecting the qualifying rate and the coefficient of variation of the hole spacing. The adjustment angle has an effect on the performance of the seed metering device (**Figure 8**). If the adjustment angle is too tiny, the time necessary for seeds to enter the hole is insufficient, affecting sowing accuracy.
2) As shown in the range analysis (**Figure 9**), if the parameters were selected from the perspective of a single factor and a single index, the single performance evaluation index is not optimal under the optimal structural parameters. As a result, the sowing performance of seed metering devices should be evaluated using a multi-factor and multi-index methodology.
3) As shown by the analysis of variance (**Figure 10**), the coefficient of variation of seed number has a significant effect(P< 0.001) when the diameter of the hole was 12 mm and 16 mm. Different hole diameters have a significant effect on the average number of holes (P*<* 0.001). There are extremely significant differences in the number of holes and average hole spacing among types (P< 0.001). The adjustment angles were 60° and 80° degrees, and the average hole spacing had a distinct effect (P< 0.001).
4) Although if the qualification rate of seeds per hole is lower than that of the fourth group, the coefficient of variation of seed number and hole spacing is better, according to the findings of the verification test. The performance of a seed metering device is often determined by multiple indexes, and a single index does not always indicate superior performance. Thus, this also demonstrates the necessity and significance of this research.
5) The maximum number of seeds per hole under the optimal parameter combination was 11, signifying that the seed metering mechanism described in this research can plant 11 seeds with a sphericity of 54.14% per hole. According to the agronomic requirements of rice direct seeding, the seed metering device designed in this study can achieve an accurate adjustment of 37~92 kg/hm^2^, and the adjustment range is superior to the rice precision seed metering device now on the market.
6) This experiment cannot accurately represent the adaptability of the seed metering device, thus many other types of spherical rice seeds will be chosen to explore adaptation further forward. Moreover, the coupling simulation method utilized in this experiment may expedite the development cycle for seed metering device research. Simultaneously, the multi-factor and multi-index optimization approach that this study presents will be helpful to future researchers studying other relevant parameter optimization.
7) The existing research methods mainly have three evaluation indexes, while this study selects six evaluation indexes. The more evaluation indexes, the more complex the optimization process becomes. The results obtained using this research method are superior to the existing relevant studies. This reflects the reliability of this research and highlights the necessity and importance of this research. The research methods and ideas are not limited to the rice direct seeding device, but can also be applied to optimizing parameters of other devices. Using this method has a significant effect on improving device performance and can improve intelligent plant protection.

## 6 Conclusion

Aiming at the problem of an unreasonable configuration of structural parameters optimization method of rice precision direct seeding metering device, a structural optimization method of rice direct seeding apparatus based on the multi-index orthogonal experiment was proposed in this paper. Firstly, the influencing factors and levels of the overall performance of the rice direct seeding device were assessed using the coupling simulation analysis method. Second, a test platform was constructed using the electric drive control model, and orthogonal tests based on numerous indices were created. Finally, a multi-index comprehensive optimization theoretical model based on the matrix analysis method was established, through which the optimal structural parameters of the seed metering device were determined, and the accuracy and qualitativeness of the optimization method of the seed metering device structure based on the multi-index orthogonal test was verified. The following are the main conclusions of this study.

1) On the basis of the multi-index orthogonal test, a thorough optimization method for the structural parameters of the seed metering device was proposed. Experiments have confirmed the suggested method. The method combines theory with co-simulation, designs the test platform using the electric drive exact control theory model, and performs the test while avoiding precise control of the speed of the seeding device in the real operation by depending on the mechanical drive.
2) The single-factor simulation test demonstrates that the seed metering device construction parameters have an effect on the performance evaluation index, and the orthogonal test parameter range was identified. From the results of a multi-index orthogonal test, the hole diameter has the most effect on the complete assessment index, while the adjustment angle has the lowest impact.
3) Following the multi-index comprehensive optimization method, the optimal factor level combination of the seed metering device’s structural parameters was A_1_B_1_C_3_. In other words, the hole’s diameter was 12 mm, the number of holes was 10, and the adjustment angle was 80°. The verification tests showed that the qualification rate of seeds per hole, empty hole rate, average seed number, coefficient of variation of seed number, average hole spacing, and variance coefficient of hole spacing are 93.07%, 0%, 9.39,14.04%, 22.84 cm, and 9.14%, respectively, under the optimal structural parameters. These results have confirmed the accuracy of the proposed method.
4) The existing research on rice direct seeding metering devices shows that the seed qualification rate per hole is less than 90%, the empty hole rate is over 3%, and the coefficient of variation of hole spacing is over 10%. The optimization method used in these studies typically focuses on no more than three evaluation indicators (Zhang et al., 2015; Zhang et al., 2017; Zhang et al., 2018a; Zhang et al., 2018b; Shun et al., 2020). However, in this study, a new method was proposed that used six evaluation indexes to optimize the parameters. The results showed a seed qualification rate per hole of 93.07%, an empty hole rate of 0%, and a coefficient of variation of hole spacing of 9.14%. These results were better than those of previous studies, highlighting the importance of this research.
5) Most of the seed metering devices currently available have 8 holes, while the device studied in this research has 10 holes. The results indicate that when seeding with the same hole spacing, the seed metering device with 10 holes has a lower rotational speed. The lower the rotational speed, the smaller the impact on the device, and the more stable its performance.
6) At present, a comprehensive optimization method of structural parameters based on a multi-index orthogonal test has been provided, which has been validated in a rice precision direct seeding metering system, and its application must be confirmed. The test method needs further improvement. For a more exact analysis, it is also important to enhance the test conditions on the bench, a challenge for future study. This approach enhances research in the area of detailed optimization of the structural parameters of a precision rice metering device under multiple indexes. The concepts and findings of this study provide techniques and references for optimizing the parameters of a precision rice seed metering device.

## Data availability statement

The raw data supporting the conclusions of this article will be made available by the authors, without undue reservation.

## Author contributions

Conceptualization, HaL, LL, and BY. Methodology, HaL, CW, and GW. Validation, HaL, ZM, and BY. Formal analysis, HaL, LL, and BY. Investigation, HuL, ZM, XA, and BY. Resources, LL, HaL, and GW. Writing—original draft preparation, HaL, CW, and BY. Writing—review and editing, HaL, and BY. Supervision, HuL, XA, and ZM. Funding acquisition, XA, ZM, and BY. All authors contributed to the article and approved the submitted version.
